# Provings of the X Ray*Read before The International Hahnemannian Association, June 30th, 1897.

**Published:** 1897-08

**Authors:** B. Fincke

**Affiliations:** Brooklyn, N. Y.


### PROVINGS OF THE X RAY.*

BY THE BROOKLYN HAHNEMANNIAN UNION.

B. FINCKE, M. D., BROOKLYN, N. Y.

A very able description of the discovery of the X ray by Prof. Roentgen was given in THE HOMŒOPATHIC PHYSICIAN (March, 1896) by our Vice-President, Dr. Walter M. James, and concluded with the words, "Why should not the homœopathist seek to procure a proving of the effects of the X ray upon the animal economy of the human being? An inviting field is thus opened up to the experimenters of our school. May it soon be cultivated."

Why not?

1897, March 27th, a drachm vial filled with absolute alcohol was exposed to a Crooke's tube in operation for half an hour, and then brought up to the sixth centesimal potency. Of this smallest globules were moistened and the vials containing them presented to the members of the Brooklyn Hahnemannian Union, which met the same evening in regular session.

Of course, every one was curious to try the new remedy and took a small number of globules on the tongue at once, and lo! it began to reveal its existence immediately, and during the two hours of our being together a number of symptoms were observed, which were at once announced and

---

* Read before The International Hahnemannian Association, June 30th, 1897.

taken down by our diligent Secretary. Some of the members from that time have been so seriously affected that they would not for any consideration try any more of that mysterious power. The following is a narrative of its action upon the several provers, being perfectly healthy up to that time, which lasted from the beginning to two months. The action reveals not only new symptoms, but also hunted up many old ones, which called forth the remark of Dr. John B. Campbell: "I noticed a grand characteristic in all the provings as far as heard from, viz., a tendency to resurrect old symptoms, some unheard of for as much as thirty years, some twenty, or a year or two."

If it is assumed that glass is opaque to the X ray, it was not so in our case, for the alcohol in the vial was certainly affected by it, as the result of the provings show. If the X ray penetrates certain tissues of the body in order to make the condition of the opaque organs, such as the bones and foreign bodies contained in the body, visible, it also shows its penetration into that invisible interior of the human being which is under the dominion of the life-force.

But oh! where is the bacillus?

The Provers were:

1. Dr. B. L. B. B. X ray 6th cent., one dose.
2. Dr. Mrs. A. B. C. Same potency, one dose.
3. Dr. J. B. C. Same potency, three doses, ten minutes apart.
4. Dr. Miss C. Same potency, one dose.
5. Dr. S. C. Same potency, one dose.
6. Mrs. E. C. Same potency, one dose.
7. Dr. L. M. L. Same potency, one dose.
8. Dr. F. H. L. Same potency, one dose.
9. Dr. Miss R. Same potency, one dose.
10. Mr. D. S. Same potency, one dose.

